# GPAQ-R: development and psychometric properties of a version of the General Practice Assessment Questionnaire for use for revalidation by general practitioners in the UK

**DOI:** 10.1186/1471-2296-14-160

**Published:** 2013-10-20

**Authors:** Martin Roland, Martin Roberts, Valerie Rhenius, John Campbell

**Affiliations:** 1 Cambridge Centre for Health Services Research, University of Cambridge, Cambridge, UK; 2 University of Plymouth Peninsula Schools of Medicine and Dentistry, Plymouth, UK; 3 CMI Publishing Ltd, Cambridge, UK; 4 University of Exeter Medical School, Exeter, UK

## Abstract

**Background:**

The General Practice Assessment Questionnaire (GPAQ) has been widely used to assess patient experience in general practice in the UK since 2004. In 2013, new regulations were introduced by the General Medical Council (GMC) requiring UK doctors to undertake periodic revalidation, which includes assessment of patient experience for individual doctors. We describe the development of a new version of GPAQ – GPAQ-R which addresses the GMC’s requirements for revalidation as well as additional NHS requirements for surveys that GPs may need to carry out in their own practices.

**Methods:**

Questionnaires were given out by doctors or practice staff after routine consultations in line with the guidance given by the General Medical Council for surveys to be used for revalidation. Data analysis and practice reports were provided independently.

**Results:**

Data were analysed for questionnaires from 7258 patients relating to 164 GPs in 29 general practices. Levels of missing data were generally low (typically 4.5-6%). The number of returned questionnaires required to achieve reliability of 0.7 were around 35 for individual doctor communication items and 29 for a composite score based on doctor communication items. This suggests that the responses to GPAQ-R had similar reliability to the GMC’s own questionnaire and we recommend 30 completed GPAQ-R questionnaires are sufficient for revalidation purposes. However, where an initial screen raises concern, the survey might be repeated with 50 completed questionnaires in order to increase reliability.

**Conclusions:**

GPAQ-R is a development of a well-established patient experience questionnaire used in general practice in the UK since 2004. This new version can be recommended for use in order to meet the UK General Medical Council’s requirements for surveys to be used in revalidation of doctors. It also meets the needs of GPs to ask about patient experience relating to aspects of practice care that are not specific to individual general practitioners (e.g. receptionists, telephone access) which meet other survey requirements of the National Health Service in England. Use of GPAQ-R has the potential to reduce the number of surveys that GPs need to carry out in their practices to meet the various regulatory requirements which they face.

## Background

Patient experience surveys have increasingly been used to assess the quality of care in general practice. In the UK, these were first used on a wide scale as part of the Quality and Outcomes Framework, a pay for performance scheme introduced in 2004 [1]. At the time, doctors were given a financial incentive to carry out patient surveys, and two surveys were approved for the purpose, the General Practice Assessment Questionnaire (GPAQ) [2] and Improving Practice Questionnaire (IPQ ) [3]. The development and validation of GPAQ from an earlier version of the survey (GPAS) has been described elsewhere [4-6] along with research carried out using GPAQ data [7-12].

In 2008, the financial incentive to carry out patient surveys using GPAQ and IPQ was removed following the introduction of a new national survey, the General Practice Patient Survey (GPPS) [13]. However, financial incentives attached to GPPS were subsequently withdrawn partly as a result of large random variations in the payments associated with patient experience scores [14]. In 2011, responsibility for conducting surveys was returned to practices, and practices against received payments for carrying out and acting on the results of patient surveys [15]. This time, there was no restriction on the questionnaires that practices could use, but many practices returned to using GPAQ.

In 2012 the UK General Medical Council (GMC) introduced a requirement for all doctors in the UK to undertake periodic revalidation. The supporting evidence for revalidation includes a requirement for patient experience to be assessed periodically at individual doctor level. The GMC has published its own questionnaire that can be used for revalidation [16] with associated publications on the development and validation of the survey [17-19]. However because the GMC survey has been designed to be used by all doctors (GPs and hospital doctors), it does not meet the needs of GPs for surveys in their own practices which include capturing patients’ views on a wider range of aspects of care, e.g. ease of getting appointments, ability to get through on the phone etc. A number of other questionnaires, including GPAQ-R, have been approved by the Royal College of General Practitioners for use in revalidation [20].

The GMC has published guidance on the development of surveys that would be approved for use in revalidation, [21] and we used this guidance to develop a new version of GPAQ (GPAQ-Revalidation or GPAQ-R) that would be suitable both for revalidation and for a range of other NHS purposes such as the incentive given to GPs to carry out patient surveys and engage patients in planning improvements based on the results [22]. The aim of our approach was to reduce the number of different surveys that GPs and practices might need to use. We describe the development and psychometric properties of this new instrument.

## Methods

### Development of the new questionnaire

GPAQ-R was developed from the existing current version of GPAQ (V3 [2]) with the following steps:

1. Moving the questions relating to the doctor patient consultation to the front of the questionnaire. These are the items necessary for revalidation. The purpose of this was so that the survey could be used with the front page alone if other items relating to wider practice organisation were not required. This also ensured that the instructions to the patient relating to the purpose of the survey were as close as possible to questions relating to the individual doctor’s performance.

2. Modification of some GPAQ questions on the doctor patient consultation to incorporate questions from the GMC questionnaire This ensured that GPAQ-R addressed the values and principles set out in the GMC’s *Good Medical Practice*[23] in a similar way to that in the GMC’s own questionnaire. Mapping of questions in GPAQ-R to *Good Medical Practice* and to the GMC’s questionnaire is shown in Table 1.

3. Cognitive testing of the new questionnaire for ease of completion and comprehensibility. This was done by VR with 10 patients with ages ranging from 18 to 90. The main aim of cognitive testing was to ensure that the questions and scales were clear and unambiguous. Only limited cognitive testing was required as all items were taken or adapted from existing questionnaires.

4. As required in GMC guidance, all relevant items include a ‘not applicable’ or ‘does not apply’ option and space was provided for free text comments on the doctor. Appropriate instructions were given about the purpose of the questionnaire and the anonymity of responses (Additional file 1)

**Table 1:** **Mapping GPAQ-R items to attributes of *Good Medical Practice *** **and to the GMC’s patient questionnaire**

**GMC framework for appraisal and revalidation: key elements for**			
**inclusion in patient questionnaires**			
**Attribute of *Good Medical***	**Examples of attribute**	**Relevant item from GMC**	**Relevant item from GPAQ-R**
***Practice***		**questionnaire**	
Developing and maintaining your professional performance	Keep knowledge and skills up to date	I am confident in this doctor’s ability to provide care	Q5. Assessing your medical condition Q8. Providing or arranging treatment for you
Applying knowledge and experience to clinical practice	Adequately assess the patient’s condition	Assessing your medical condition	Q5. Assessing your medical condition
	Support patients in caring for themselves	Involving you in decisions about your treatment. Explaining your condition and treatment	Q7. Involving you in decisions about your care Q6. Explaining your condition and treatment
	Work within the limits of your competence.	How good was the doctor at providing or arranging treatment for you.	Q8. Providing or arranging treatment for you
	Provide or arrange advice, investigations, treatment. Prescribe drugs appropriately		
Keeping personal information securely	Implement and comply with systems to protect patient confidentiality	The doctor will keep the information about me confidential	Q10. Confident that the doctor will keep your information confidential
Communicating effectively	Listen to patients and respecting their views	Listening to you	Q3. Listening to you
	Give patients the information they need	Explaining your condition and treatment	Q6. Explaining your condition and treatment
	Respond to patients’ questions		Q3. How good was the GP at listening to you?
Showing respect for patients	Implement and comply with systems to protect patient confidentiality	The doctor will keep the information about me confidential	Q10. Confident that the doctor will keep your information confidential
	Be polite and considerate and respect patient’s dignity and privacy	Being polite, making you feel at ease	Q1. Putting you at your ease Q2. Being polite and considerate Q4. Giving you enough time
Acting with honesty and integrity	Being honest	This doctor is honest and trustworthy	Q9. Confidence that the GP is honest and trustworthy

We did not undertake specific consultation with patient or professional groups in developing this version of GPAQ. Items for inclusion in GPAQ were originally based on systematic reviews of aspects of care that are important to patients, updated by a more recent systematic review by one of the authors [24]. In addition, GPAQ questions have been modified over the past 10 years based in part on feedback from patient and practice groups which have used the questionnaire as part of the Quality and Outcomes Framework.

The final tested version of GPAQ-R is shown in Additional file 2. Updated versions and conditions for use are available at http://www.gpaq.info. In general, GPAQ-R is freely available for individual practices to download and use, but commercial organisations may not use GPAQ without a license.

Practices were instructed to give questionnaires to consecutive patients attending practices in line with GMC guidance on the completion of surveys (instructions to practices are given in Additional file 1). The questionnaires were administered by practice staff but the completed questionnaires were not seen by practice staff and all analyses were carried out independently. Surveys were carried out for GPs who were partners or salaried doctors but trainees were not included. We chose to administer the questionnaires in the way in which they would be used for revalidation in line with GMC guidance and therefore we do not have details of response rates or whether any patients were deliberately excluded from the survey. Data were supplied by CMI Publishing Ltd and Intime Data, two commercial firms with licenses from the University of Cambridge to supply GPAQ services to practices.

### Statistical analysis

We described the demographic profile of the patient sample, the frequency distributions of responses to the eleven core doctor-patient communication and confidence items (Q1 to Q11) that related to the elements required by the GMC for revalidation, and the rates of missing or spoilt responses to these items. Valid responses to these items were scored linearly from 0 (least favourable) to 100 (most favourable) ignoring ‘Doesn’t apply’ and ‘Don’t know’ responses. No attempt was made to impute missing values. We calculated the reliability of these core item scores from the intraclass correlation coefficients (ICCs) and estimated the number of patient responses needed to achieve 0.7 or 0.8 reliability for the doctor’s mean score on each item.

We conducted an exploratory factor analysis for doctors where data were complete on the eleven core items. The analysis used principal components extraction, applying a Varimax rotation with Kaiser normalisation to improve the interpretability of the solution and retaining factors with eigenvalues greater than one.

For each of the doctors with at least six patient questionnaires ‘communication’ and ‘confidence’ scores were calculated as follows. We averaged each of the communication items (Q1 to Q8) and confidence items (Q9 to Q11) across all patients rating the doctor, provided that at least six valid patient responses for that doctor were present. Finally we averaged the mean communication item scores and averaged the mean confidence item scores provided, in each case, that more than half of them were present. The reliabilities of the communication and confidence scores were evaluated using Generalisability Theory [25].

All analyses were conducted in SPSS version 20 except for the generalisability analysis which used G_String_IV. No attempt was made to impute missing values except in the generalisability analysis where missing item scores were replaced by the grand mean in line with accepted practice for generalisability analysis [26].

## Results

Data were analysed for questionnaires from 7258 patients relating to 164 GPs in 29 practices (mean 44 responses per GP). The majority of respondents (70.8%) were under 65, with 17.1% and 12.1% of respondents 65–74 and 75 or over respectively. Most (64%) were female and 55.4% recorded that they had a long-standing health condition. 90.9% of respondents recorded their ethnicity as ‘White’ and just under half (47%) were currently in employment.

Table 2 shows the frequency distribution of responses to the questions on communication with the doctor which were the core questions relating to the GMC criteria for revalidation. Responses were, as expected, skewed with many more positive than negative responses. Rates of missing or spoilt responses to the core questions varied between 4.5% and 6.0%, except question 11 (‘Would you be completely happy to see this GP again?’) where only 87.8% of patients recorded valid responses. ‘Spoilt’ responses included those where the patient had recorded free text instead of checking one of the boxes, but also included data entry errors.

**Table 2:** Frequency distributions for GPAQ doctor communication items

**Item**	**Number**		**Number of valid responses**	**Very good**	**Good**	**Satisfactory**	**Poor**	**Very poor**	**Does not apply**
	**missing or spoilt**								
	**%**			**%**	**%**	**%**	**%**	**%**	**%**
How good was the GP at:									
Q1 Putting you at ease?	328	(4.5)	6930	79.6	16.3	3.5	0.2	0.1	0.2
Q2 Being polite and considerate?	332	(4.6)	6926	85.4	12.3	1.9	0.2	0.0	0.1
Q3 Listening to you?	341	(4.7)	6917	82.4	14.6	2.5	0.3	0.1	0.1
Q4 Giving you enough time?	345	(4.8)	6913	76.0	18.6	4.5	0.6	0.1	0.2
Q5 Assessing your medical condition?	356	(4.9)	6902	74.5	19.1	4.8	0.4	0.1	1.1
Q6 Explaining your condition and treatment?	351	(4.8)	6907	72.3	20.4	4.8	0.4	0.1	2.0
Q7 Involving you in decisions about your care?	399	(5.5)	6859	69.2	20.8	5.5	0.3	0.1	4.0
Q8 Providing or arranging treatment for you?	439	(6.0)	6819	73.4	16.8	3.9	0.3	0.2	5.4
				Yes, definitely %	Yes, to some extent%	No, not at all%	Don’t know / can’t say%		
Q9 Did you have confidence that the GP is honest and trustworthy?	362	(5.0%)	6896	94.0	5.0	0.3	0.7		
Q10 Did you have confidence that the doctor will keep your information confidential?	391	(5.4%)	6867	95.2	3.2	0.1	1.5		
				Yes%	No%				
Q11 Would you be completely happy to see this GP again?	886	(12.2.%)	6372	99.1	0.9				

N = 7258.

The factor analysis (Table 3) used data from 5,569 patients with complete data on the eleven core items. A two-factor solution (Table 4) explained 66% of the total variance, relating to communication (Qs 1–8, 56% of the variance) and trust / confidence (Qs 9–11, 10% of the variance), with eigenvalues of 6.154 and 1.120 respectively. These correspond to the results of factor analysis previously reported for the GMC patient questionnaire [18].

**Table 3:** Factor analysis of survey items required by the General Medical Council for revalidation

	**Component**	
	**1**	**2**
Q5 Doctor: Assessing your medical condition?	0.832	
Q7 Doctor: Involving you in decisions about your care	0.820	
Q3 Doctor: Listening to you?	0.818	
Q6 Doctor: Explaining your condition and treatment	0.817	
Q4 Doctor: Giving you enough time?	0.817	
Q1 Doctor: Putting you at ease?	0.806	
Q2 Doctor: Being polite and considerate?	0.777	
Q8 Doctor: Providing or arranging treatment for you	0.770	
Q10 Did you have confidence that the doctor will keep your information confidential?		0.806
Q9 Did you have confidence that the GP is honest and trustworthy?	0.347	0.716
Q11 Would you be completely happy to see this GP again?		0.608

Loadings less than 0.3 are omitted from the table.

**Table 4:** Mean score (SD) and reliability of GPAQ doctor communication items

**Outcome measure**	Mean*	**(SD)**	**ICC**	**Responses for**	**Responses for**
				**0.70 reliability**	**0.80 reliability**
How good was the GP at:					
Q1 Putting you at ease?	93.9	(13.2)	0.074	30	50
Q2 Being polite and considerate?	95.7	(11.1)	0.059	38	64
Q3 Listening to you?	94.8	(12.2)	0.060	37	63
Q4 Giving you enough time?	92.6	(14.7)	0.056	40	68
Q5 Assessing your medical condition?	92.3	(14.7)	0.052	43	74
Q6 Explaining your condition and treatment?	91.9	(14.9)	0.066	34	58
Q7 Involving you in decisions about your care?	91.3	(15.4)	0.067	33	56
Q8 Providing or arranging treatment for you?	93.1	(14.2)	0.066	34	58
Q9 Did you have confidence that the GP is honest and trustworthy?	97.2	(12.2)	0.053	42	72
Q10 Did you have confidence that the doctor will keep your information confidential?	98.2	(9.6)	0.032	71	121
Q11 Would you be completely happy to see this GP again?	99.1	(9.5)	0.007	326	558

*All responses were scored linearly from 0 (least favourable) to 100 (most favourable) ignoring ‘Doesn’t apply’ and ‘Don’t know’ responses

ICC = intra-class correlation coefficient

Table 4 shows the intra-class correlations (ICCs) for the core items along with the number of patient responses per doctor needed to achieve 0.7 or 0.8 reliability for the doctor’s mean score for each item. These results suggest that around 35 responses (range 30 to 43 for individual items) would be needed to give reliability of 0.7 on individual communication items.

148 doctors provided data allowing calculation of a mean score for both communication and confidence. The mean (SD) of the mean communication scores was 92.8 (3.90) and of the confidence scores was 98.0 (2.15). Variance component analysis of the communication score identified that 58% of variance was attributable to patients, whilst only 5% was attributable to doctors, and only 1% to items. Corresponding figures for the confidence score were 28%, 2% and 1% respectively. Reliability is thus best improved by increasing the number of patients returning the questionnaire rather than by varying the number of items in the questionnaire. The generalisability analysis showed that a generalisability coefficient (reliability) of 0.70 can be achieved for the communication score with 29 patient questionnaires per doctor, or of 0.8 with 50 questionnaires per doctor. The confidence score was less reliable: the corresponding figures were 81 and 191 questionnaires respectively.

## Discussion

The results suggest that GPAQ-R, a development of previous versions of the General Practice Assessment Questionnaire, is suitable to use for revalidation of doctors in the National Health Service, meeting the requirements for survey development set out by the General Medical Council and with psychometric properties similar to those of the GMC’s own questionnaire [17]. We recommend that 30 completed questionnaires should be obtained to give sufficiently reliable results for scoring doctor-patient communication for individual doctors. However, where this initial screen raises concern, a survey might be repeated with 50 returned questionnaires to give greater reliability, increasing the reliability coefficient from 0.7 to 0.8.

While these numbers give satisfactory levels of reliability for both items and the composite scale for doctor patient communication, they do not for the scale on trust and confidence or for two out of the three individual items on trust and confidence. In particular, for the item ‘Would you be completely happy to see this GP again?’ where over 99% of patients replied ‘Yes’, over 300 responses would be needed to achieve reliability of 0.7. Although taken from the GMC questionnaire, this item is unlikely to be discriminating as a screen for poor performance.

The strengths of this questionnaire compared to the GMC’s questionnaire are that it intentionally incorporates a range of practice characteristics to be assessed and is therefore suitable for a wider range of uses within the NHS than the GMC questionnaire which focuses solely on items relevant to revalidation. However, because of this, GPAQ-R is also considerably longer than the GMC questionnaire and this could affect response rate. It is important to note that the GMC’s recommended methodology (handing out questionnaires after a consultation) does not require response rates to be recorded. For GPs only wishing to use GPAQ-R for revalidation purposes, we have designed the survey so that the front page can be used on its own, which significantly shortens the questionnaire.

The relatively high non-completion rate for one item is of concern, namely the 12% of patients who did not provide valid responses to the question “Would you be completely happy to see this GP again?”, although some of these were data entry errors. We do not think this is due to wording of the question as the phrasing of this item is virtually identical to the GMC’s own questionnaire where lower non-complete rates have been reported. The high non-completion rate for this item may be in part due to the proximity of space for patients to make free comments about their experience with the GP. Thirty five of the blank items on this question had associated handwritten comments and we have now modified the instruction on the first page to include a comment on the importance of completing all questions. Patients may also be concerned that doctors would see the response to this question, and we note that GMC guidance is that patients should return questionnaires in a sealed envelope which may increase their confidence in that their answers will remain confidential, and we are not certain that this guidance was always followed in this study. Where patients choose to give a free text comment as an alternative to ticking a box, we believe that this is likely to indicate that the patient regards this as more valuable information, and we have adopted this approach in other research which we are carrying out. We therefore recommend that free text comments should form part of the feedback that doctors receive on their performance. However, if this is done, some comments need to be anonymised before being fed back which substantially increases the costs of processing the questionnaire data.

GPAQ-R, like the GMC questionnaire, takes an approach of asking about the quality of communication (e.g. ‘How good was the doctor at ….’), sometimes called evaluation questions. This contrasts with some other surveys which focus on whether particular questions were asked (e.g. ‘Did the doctor ask you about …’), sometimes called report questions which are sometimes regarded as less subjective and easier to interpret [27]. A commonly cited cognitive model of how patients respond to questionnaire items was developed by Tourangeau [28] who suggests that completion of survey questions requires (1) comprehension of the question, (2) retrieval from memory of the relevant information, (3) use of the information to make a judgment if the question calls for one, and (4) selection and reporting of the response. Although report and evaluation approaches are sometimes contrasted, we believe that the difference between the two is modest provided very specific questions are asked, partly because ‘report’ items often have an evaluative component implied in their wording and in many circumstances both require a judgement to be made (stage 3 of Tourangeau’s model). Items in GPAQ-R ask for the patient’s evaluation of very specific aspects of care and do not include questions on general satisfaction.

## Conclusions

GPAQ-R is a development of a well-established patient experience questionnaire used in general practice in the UK since 2004. This new version can be recommended for use in order to meet the UK General Medical Council’s requirements for surveys to be used in revalidation of doctors. It also meets the needs of GPs to ask about patient experience relating to aspects of practice care that are not specific to individual general practitioners (e.g. receptionists, telephone access) which meet other survey requirements of the National Health Service in England. Use of GPAQ-R has the potential to reduce the number of surveys that GPs need to carry out in their practices to meet the various regulatory requirements which they face.

### Ethical consent

Ethical permission was not required for these analyses.

## Competing interests

Professor Roland and Professor Campbell developed the original version of GPAQ in 1998. Between 2005 and 2008, the University of Manchester received payments from the licensing of GPAQ to commercial suppliers who provided a service to run surveys for general practices in England. The University of Cambridge now receives these payments. Valerie Rhenius owns one of the licensed suppliers (CMI Publishing Ltd). Ms Rhenius and the University of Cambridge therefore have the potential for to gain financially from widespread use of GPAQ-R.

## Authors’ contributions

MRol and JC designed the study. VR provided data from her own company and another licensee. The statistical analysis was carried out principally by MRob. All authors contributed to writing and revising drafts of the paper. All authors read and approved the final manuscript.

## Pre-publication history

The pre-publication history for this paper can be accessed here:

http://www.biomedcentral.com/1471-2296/14/160/prepub

## Supplementary Material

Additional file 1 Instructions to practices for carrying out surveys. Click here for file

Additional file 2 General Practice Assessment Questionnaire for Revalidation (GPAQ-R). Click here for file
