# Supplementary material for: GPAQ-R: development and psychometric properties of a version of the General Practice Assessment Questionnaire for use for revalidation by general practitioners in the UK
Source: BMC Fam Pract. 2013 Oct 20;14:160. doi: 10.1186/1471-2296-14-160 (PMC3819733; doi:10.1186/1471-2296-14-160)
Supplement: Additional file 1 — Instructions to practices for carrying out surveys. [file 1471-2296-14-160-S1.doc]

**Additional file 1. Instructions to practices for carrying out surveys**


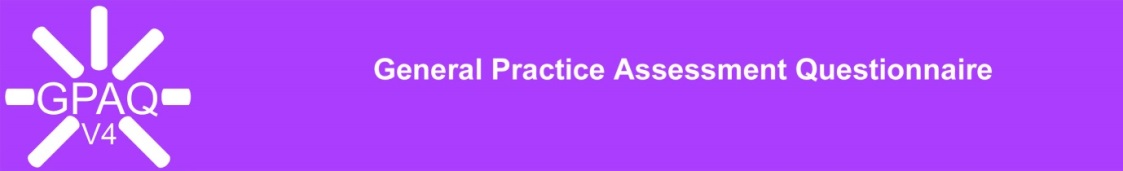


**Guidelines for carrying out your practice survey**

For revalidation purposes, the GMC requirements are very specific, and it is important to fulfil these requirements for results to be accepted.

**Preparation:**

1. Prepare a ‘postal’ box into which patients can post their completed questionnaires
2. Prepare instruction sheets for the patients, have a supply of A5 envelopes for completed questionnaires and have black pens available.
3. Fill in each doctor’s name on a blank copy of the questionnaire before making the required number of copies for each GP.
4. If you are surveying nurses only, we suggest either adapting the questionnaire, or asking patients to start at p2.

**Carrying out the Survey:**

1. Appoint someone, with sufficient allocated time, to look after the survey. They should invite successive patients for the doctor to take part in the survey when they are booking in for their appointment, and be encouraged to complete the questionnaire before leaving the surgery.
2. Ensure that 10% more questionnaires are given out than are needed for each doctor. Reporting may only be valid if the requisite numbers are achieved.
3. Give one survey form, one instruction sheet (can be recycled!) and one A5 envelope to each patient.
4. Explain that as well as the annual practice survey, doctor is seeking his/her patient’s views on how well they perform and whether they could improve the way they work.
5. Tell patients the survey should take about 10 minutes, views are confidential and should be their honest opinions.
6. Also explain that although the questionnaire seems long, it covers two bases and saves the need for an additional survey.
7. An accompanying adult can fill in the questionnaire on behalf of a child (under 12); patients with disabilities can ask for assistance; judgement may be needed where it is inappropriate to ask for feedback, if patient is too ill or distressed.

# If the GP is handing out the questionnaire

1. The GP should hand out **one** questionnaire, **one** information sheet and **one** envelope to each patient.
2. These should be handed to **every patient**, consecutively on the GP’s clinic list.
3. **All** the questionnaires should be handed out to ensure an adequate numerical response.

# Very important

The doctor must not have access to completed questionnaires. GMC requirements for revalidation are that questionnaires must be sealed in an envelope before posting into a box supplied by the surgery.
