# Supplementary figures and images for: GPAQ-R: development and psychometric properties of a version of the General Practice Assessment Questionnaire for use for revalidation by general practitioners in the UK
Source: BMC Fam Pract. 2013 Oct 20;14:160. doi: 10.1186/1471-2296-14-160 (PMC3819733; doi:10.1186/1471-2296-14-160)

**Additional file 2. General Practice Assessment Questionnaire for Revalidation (GPAQ-R)**

**
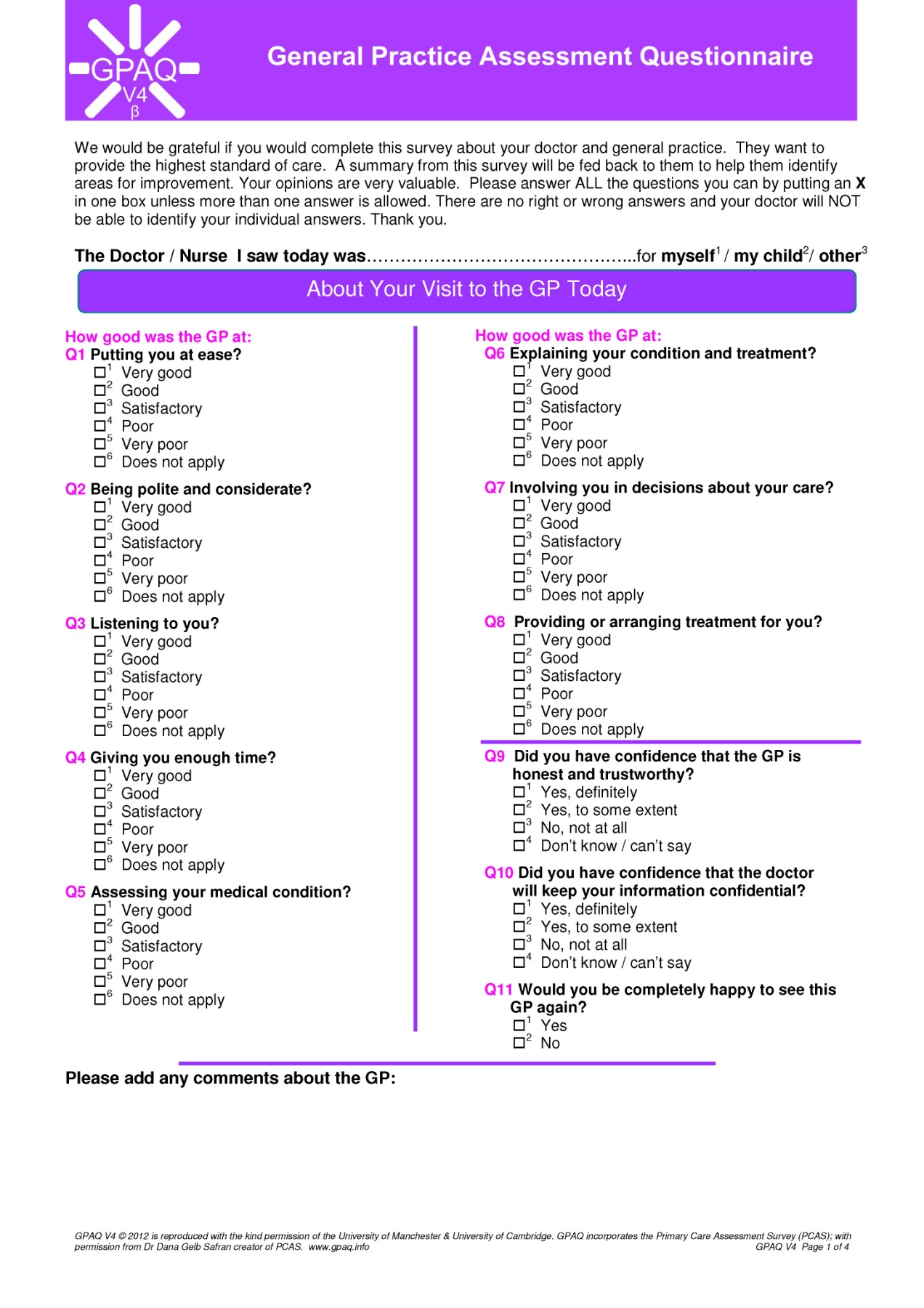
**


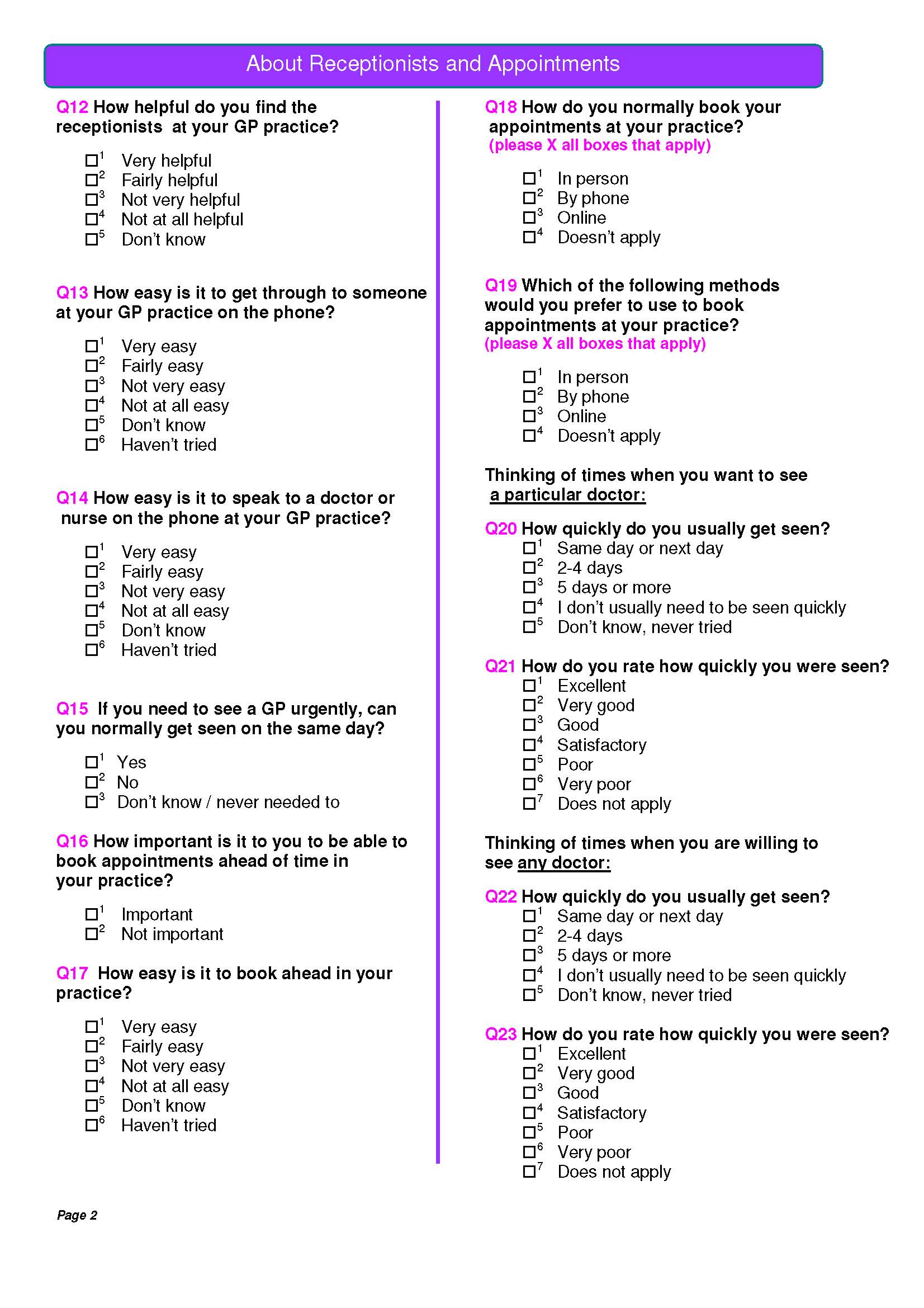


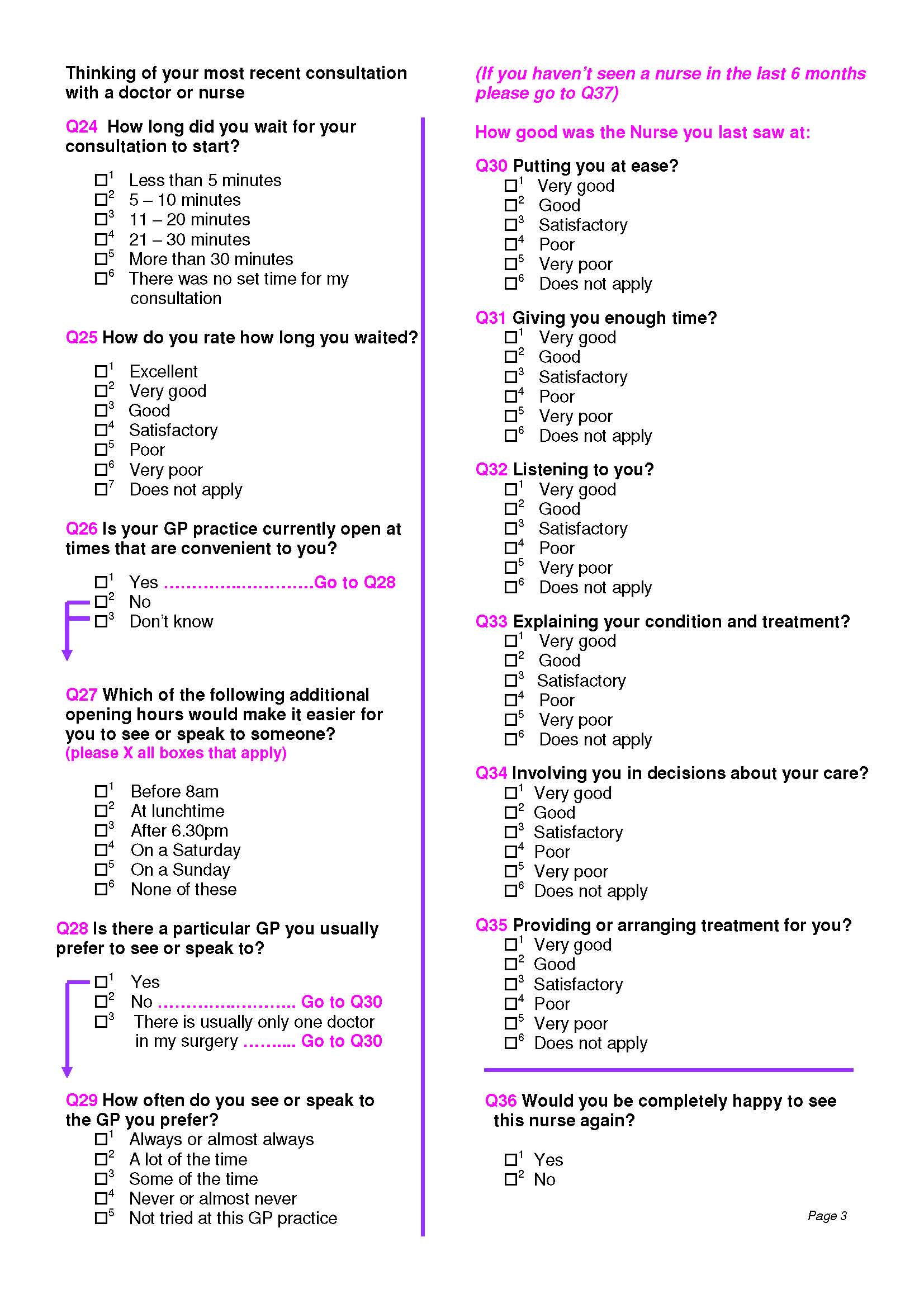


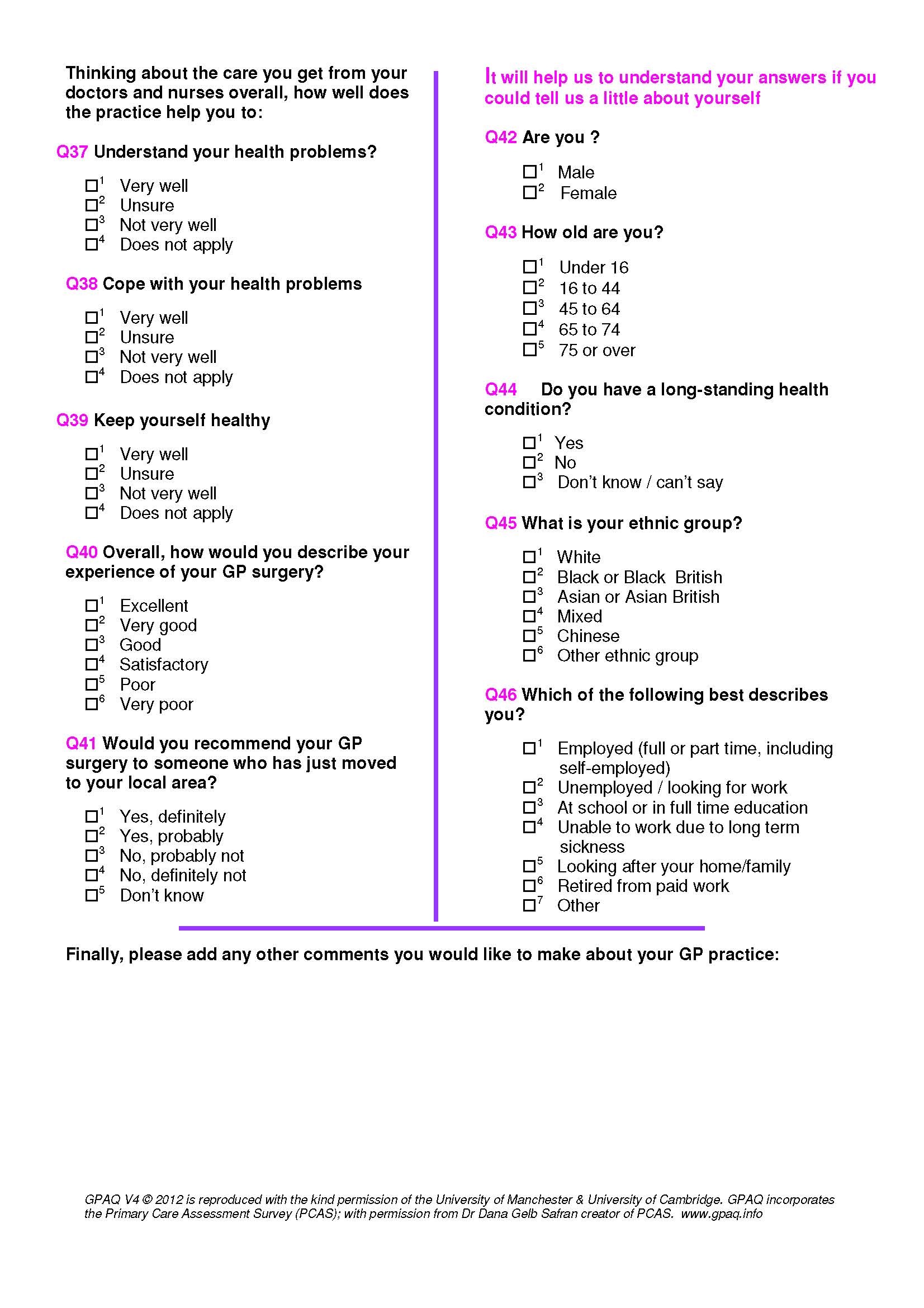

Supplement: Additional file 2 — General Practice Assessment Questionnaire for Revalidation (GPAQ-R). [file 1471-2296-14-160-S2.doc]
